# Sero-prevalence and factors associated with *Toxoplasma gondii* infection among pregnant women attending antenatal care in Mwanza, Tanzania

**DOI:** 10.1186/1756-3305-6-222

**Published:** 2013-08-06

**Authors:** Berno Mwambe, Stephen E Mshana, Benson R Kidenya, Anthony N Massinde, Humphrey D Mazigo, Denna Michael, Charles Majinge, Uwe Groß

**Affiliations:** 1 Department of Obstetrics and Gynaecology, Catholic University of Health and Allied Sciences, Box 1464, Mwanza, Tanzania; 2 Department of Microbiology/Immunology, Catholic University of Health and Allied Sciences, Box 1464, Mwanza, Tanzania; 3 Department of Biochemistry and Molecular Biology, Catholic University of Health and Allied Sciences, Box 1464, Mwanza, Tanzania; 4 Department of Parasitology, Catholic University of Health and Allied Sciences, Box 1464, Mwanza, Tanzania; 5 Mwanza Research Centre, National Institute of Medical Research Mwanza, Mwanza, Tanzania; 6 Institute of Medical Microbiology and German Consulting Laboratory for Toxoplasma, Göttingen University Medical Center, Göttingen, Germany

## Abstract

**Background:**

Serological screening of pregnant women for *Toxoplasma gondii*-specific antibodies is not practiced as an antenatal care in Tanzania; and there is a limited data about sero-prevalence of *T. gondii* infection in developing countries. We therefore conducted this study to determine the sero-prevalence and factors associated with *T. gondii* infection among pregnant women attending antenatal care clinics in Mwanza, Tanzania.

**Methods:**

Between 1^st^ November 2012 and 31^st^ May 2013 a total of 350 pregnant women attending antenatal care clinics in Mwanza were enrolled and screened for IgG and IgM antibodies against *T. gondii* using the ELISA technique.

**Results:**

Of 350 pregnant women, 108 (30.9%) were sero-positive for *T. gondii*-specific antibodies. The risk of contracting *T. gondii* infection increases by 7% with each yearly increase in a woman’s age (OR=1.07, 95% CI: 1.02 - 1.11, p=0.002). The sero-positivity rate of *T. gondii-*specific antibodies was higher among pregnant women from the urban than those from rural communities (41.5% versus 22.0%); [OR=2.2, 95% CI; 1.4 - 3.7, p=0.001]. Likewise employed/business women were more likely to get *T. gondii* infection than peasants (40.0% versus 25.9%) [OR=1.9, 95% CI: 1.2 - 3.0, p=0.006].

**Conclusions:**

Sero-prevalence of *T. gondii-*specific antibodies is high among pregnant women in Mwanza with a significant proportion of women at risk of contracting primary *T. gondii* infections. Screening of *T. gondii* infections during antenatal care should be considered in Tanzania as the main strategy to minimize congenital toxoplasmosis.

## Background

Primary infections with *T. gondii* acquired during pregnancy are usually asymptomatic for the pregnant woman but can lead to serious neonatal complications [1]. Toxoplasmosis in pregnancy has been associated with miscarriage, hydrocephalus, cerebral calcification and chorioretinitis in the newborn [2]. Primary infection with *T. gondii* during the third trimester of pregnancy carries a higher risk of congenital transmission than if it is acquired during the first trimester [3,4]. Generally, it is estimated that about one third of the world’s population is infected with *T. gondii*[5,6]. In developed countries, congenital toxoplasmosis affects 0.01% - 0.1% of infants [5]. In a study done in Dar es Salaam, Tanzania a sero-prevalence of 35% was reported in pregnant women in 1995 [7]. Since then there has been no follow-up study done and no intervention programme implemented. Antenatal serological screening of *T. gondii* infection based on IgG and IgM detection is the mainstay in monitoring the risk for congenital toxoplasmosis. Maternal-fetal intervention for toxoplasmosis can be achieved through the use of drugs such as spiramycine which prevents congenital infection by more than 60% [5]. In Tanzania, there is no screening program during antenatal care for pregnant women. Therefore, this study aimed at determining the seroprevalence of *T. gondii* infection and its associated factors to provide basic information that could be used to justify the introduction of an antenatal screening programme in Tanzania.

## Methods

An analytical cross–sectional health facility based study was conducted from 1^st^ November 2012 to 31^st^ May 2013 and was approved by Bugando Medical Centre/Catholic University of Health and Allied Sciences ethics review committee. The study was conducted in Mwanza, in the three highly populated public antenatal care clinics as representative sites (Igombe Health Centre, Sekou-Toure Regional Hospital and UMATI clinic). Igombe Health Centre is located in Ilemela district about 20 kilometers from Mwanza city centre, Igombe was considered as a rural area in this study. Sekou-Toure Regional Hospital and UMATI clinic are located in Mwanza city centre and were considered as urban areas. Mwanza is one of the largest cities of Tanzania and the capital of the Lake Victoria region. The staple foods for people living in Mwanza are maize, rice, fish, beef and chicken. The meat is usually cooked but may be eaten undercooked especially when taken in bars (Nyama Choma).

All pregnant women attended these clinics with gestational age between 8–42 weeks were serially enrolled until the sample size was reached. The sample size was estimated using the Kish Lisle formula for a cross-sectional study; a prevalence (p) of 35% from the study in Dar es Salaam was used to calculate the sample size [7], resulting in a minimum sample size of 350 pregnant women.

### Sample collection procedure and laboratory work

About 4 mL of venous blood was collected aseptically from each of the 350 pregnant women, of these 180 were from Igombe and 170 from UMATI and Sekou-Toure. All specimens were transported to the Bugando Medical Centre where serum was separated from the whole blood by centrifugation at 3,000 rpm for 5 minutes. Separated serum was numbered and kept at −80°C until transportation to Germany for further analysis at the National Consulting Laboratory of Toxoplasma. Sera were tested for anti-*T. gondii* antibodies using ELISA test kits (VIDAS Toxo-IgG-II-ELFA and Toxo-IgM-ELFA for anti-*T. gondii*-specific IgG and IgM antibodies respectively (bioMérieux GmbH, Nürtingen, Germany). The cut-off values for detection of IgG were as follows: IgG levels <4 IU/mL were negative; IgG levels 4 - <8 IU/mL were borderline and IgG levels ≥8 IU/mL were positive. The cut-off values for detection of IgM were as follows: IgM index value of <0.55 was negative; IgM index value of 0.55 - <0.65 was borderline and IgM index value of ≥0.65 was positive. Demographic data such as age, residence, occupation, marital status, education, and relevant clinical information such as gravidity and trimester were collected using a pre-tested standardized data collection tool.

### Data management and analysis

Data were entered into a computer using Microsoft Office Excel 2007 according to codes given, and analyzed using the STATA version 12 (College Station, Texas, USA). Categorical variables were summarized as proportions and were analyzed using the Pearson’s Chi-square test to observe the differences among the various groups. Continuous variables were summarized as mean ± standard deviation. Univariate analysis and multivariate logistic regression models were fitted to determine factors associated with *T. gondii* infection. These factors were age, residence, occupation, education level, gravidity, eating of undercooked meat, drinking unsafe water and contact with cats. We used a backward-stepwise selection model to select factors with a p-value of less than 0.2 to be fitted into the multivariate logistic regression analysis. Odds ratios (OR) and their and 95% confidence interval [95% CI] were noted. Factors with the p-value of less than 0.05 on multivariate logistic regression analysis were considered to have a statistically significant association with *T. gondii* infection.

## Results

**Figure 1:**
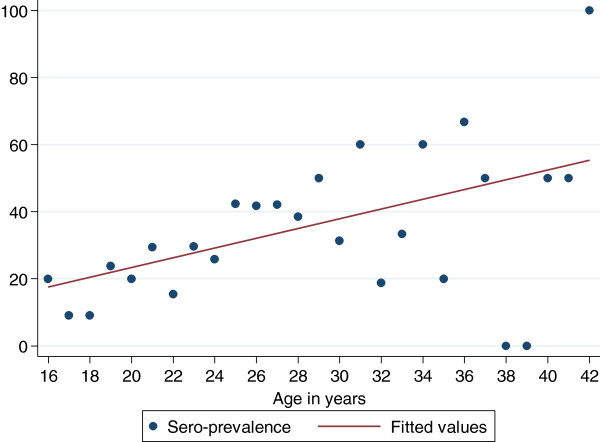
**Age specific Sero-prevalence of *Toxoplasma gondii*.** There is an increase of sero-prevalence of *T. gondii* infection with an increase in age. The sero-prevalence increases by 1.4% with one year increase in age. The risk (odds ratios) of acquiring *T. gondii* infection increases by 7% with one year increase in age.

A total of 350 pregnant women were enrolled during the study period with the mean age of 25.3 ± 5.4 years with majority of women 174/350 (49.7%) aged between 15–24 years. Most of the women were from a rural residence 186/350 (53.1%), peasants 220/350 (62.9%), married 294/350 (84.0%), multigravid 188/350 (53.7%) and on third trimester 185/350 (52.9%). The majority of women 243/350 (69.4%) had primary education, with 86/350 (24.6%) attaining a higher level of education (Table 1). Of the 350 pregnant women studied, 108 (30.9%) were positive for anti-*T. gondii*-specific IgG antibodies, indicating past infection (Table 1). None had positive IgM results. The risk of contracting *T. gondii* infection increases by 7% with each yearly increase in a woman’s age (OR=1.07, 95% CI: 1.02 - 1.11, p=0.002). The sero-prevalence increases by 1.4% with one year increase in age (Figure 1).

**Table 1:** **Distribution of *T. gondii *sero-prevalence along with demographic characteristics among pregnant women, Mwanza, 2013**

**Patients characteristic**	***T. gondii *seroprevalence (N=350)**		**Total**
	**Positive**	**Negative**	
	**n (%)**	**n (%)**	
***Age (Yr)***			
15-24	38 (21.8)	136 (78.2)	174
25-34	61 (40.1)	91 (59.9)	152
35-44	10 (41.7)	14 (58.3)	24
***Residence***			
Urban	68 (41.5)	96 (58.5)	164
Rural	41 (22.0)	145 (78.0)	186
***Occupation***			
Business	37 (37.8)	61 (62.2)	98
Peasant	57 (25.9)	163 (74.1)	220
Employed	15 (46.9)	17 (53.1)	32
***Education***			
Illiterate	7 (33.3)	14 (66.7)	21
Primary	66 (27.2)	177 (72.8)	243
Secondary and above	36 (41.9)	50 (58.1)	86
***Marital status***			
Married	89 (30.3)	205 (69.7)	294
Unmarried	20 (35.7)	36 (64.3)	56
***Gravidity***			
Primigravid	33 (29.5)	79 (70.5)	112
Multigravid	61 (32.5)	127 (67.5)	188
Grandmultigravid	15 (30.0)	35 (70.0)	50
***Trimester***			
1st Trimester	4 (26.7)	11 (73.3)	15
2nd Trimester	50 (33.3)	100 (66.7)	150
3rd Trimester	55 (29.7)	130 (70.3)	185

The sero-prevalence of *T. gondii*-specific IgG antibodies was higher in pregnant women residing in urban (41.5%) than those in rural areas (22.0%) [OR=2.2, 95% CI: 1.4 - 3.7, p=0.001] (Table 2). A total of 220/350 (62.9%) of the pregnant women were peasants, of these, 25.9% (57/220) tested positive for *T. gondii-*specific antibodies. On univariate analysis there was a significant difference in *Toxoplasma* sero-positivity among individuals with different occupations; employed/business pregnant women had a higher sero-positivity rate of *T. gondii-*specific antibodies than peasants (40.0% versus 25.9%) (OR=1.9, 95% CI: 1.2 – 3.0, p=0.006). The factor occupation of pregnant women was not subjected into multivariate analysis because it had a co-linearity relation with residence; as most of the peasants were living in rural areas.

**Table 2:** **Factors associated with *T. gondii *infection among pregnant women (N=350) in Mwanza**

**Patient’s characteristic**	***Toxoplasma *sero-prevalence**		**Univariate**		**Multivariate**	
	**Positive**	**Negative**	**OR [95% CI]**	**p-value**	**OR [95% CI]**	**p-value**
	**n (%)**	**n (%)**				
***Age (years)***						
15-24	38 (21.8)	136 (78.2)	1		1	
25-34	61 (40.1)	91 (59.9)	2.4 [1.5-3.9]	<0.001	2.2 [1.4-3.7]	0.001
35-44	10 (41.7)	14 (58.3	2.6 [1.1-6.2]	0.038	2.3 [0.9-5.7]	0.070
***Residence***						
Rural	41 (22.0)	145 (78.0)	1		1	
Urban	68 (41.5)	96 (58.5)	2.5 [1.6-4.0]	<0.001	2.2 [1.4-3.7]	0.001
***Occupation***						
Peasant	57 (25.9)	163 (74.1)	1			
Employed/Business	52 (40.0)	78 (60.0)	1.9 [1.2-3.0]	0.006	-	-
***Education***						
Illiterate	7 (33.3)	14 (66.7	1			
Primary	66 (27.2)	177 (72.8)	0.7 [0.3-1.9]	0.545	-	-
Secondary/above	36 (41.9)	50 (58.1)	1.4 [0.5-3.9]	0.476	-	-
***Unsafe water***						
Yes	62 (28.1)	159 (71.9)	1		1	
No	47 (36.4)	82 (63.6)	1.5 [0.9-2.3]	0.103	1.2 [0.7-1.9]	0.541
***Gravidity***						
Primigravid	33 (29.5)	79 (70.5)	1			
Multigravid	61 (32.5)	127 (67.5)	1.1 [0.7-1.9]	0.590	-	-
Grandmultigravid	15 (30.0)	35 (70.0)	1.0 [0.5-2.1]	0.945	-	-
***Undercooked meat***						
Yes	48 (28.4)	121 (71.6)	1			
No	61 (33.7)	120 (66.2)	1.3 [0.8-2.0]	0.285	-	-
***Contacts with Cat***						
No	64 (32.6)	132 (67.4)	1			
Yes	45 (29.2)	109 (70.8)	0.9 [0.5-1.3]	0.491	-	-

## Discussion

The sero-prevalence of *T. gondii* infection in this study was lower than that reported in Dar es Salaam, Tanzania two decades ago in which more than 35% of the pregnant women had evidence of *T. gondii* infections [7,8]. The difference could partly be explained by the geographical variation; Dar es Salaam is hotter than Mwanza and hotter weather has been found to favour sporulation of oocysts; secondly the difference could be due to the diagnostic methods, a study in Dar es Salaam used an immunosorbent agglutination assay while in the current study a highly specific ELISA method was used. However, the variation of sero-prevalence within a given country is not a new phenomenon; in USA the seroprevalence was found to vary from 17.5% in the west to 20.5% and 29.2% in the south-midwest and north-east, respectively [9]. Also the sero-prevalence in this study was low compared to studies in Brazil [10], Saudi Arabia [11], Morocco [12] and Sudan [13]. This may be accounted for by differences in climatic conditions, as reported before, where higher sero-prevalence is associated with hotter and wetter areas, which is favourable for sporulation of oocysts compared to less humid areas [14,15].

An increase in sero-positivity of anti-*T. gondii* antibodies was observed with increasing age in this study, which is consistent with other studies [6,16]. The observed risk increase per year might be considered high and may reflect higher infection risks at early adolescence. In this study, business women and employed pregnant women had higher infection rates with *T. gondii* than peasants, in contrary to other studies [6,17]. This may partly be explained by the economic status of the pregnant women in which more employed and business women, who have a high income compared to peasants; also tend to live in towns and can afford to eat poultry and pork which have been found to be a major source for *T. gondii* transmission [17,18]. This observation is further supported by the fact that, in this study, as it was observed in China [19]; residents from urban areas were more infected with *T. gondii* than those from rural areas.

Contaminated fruits and eating undercooked meat has been reported as a potential source of *T. gondii* infection [18,20-22]. Drinking contaminated water is another source of *T. gondii* infection [23,24]. A study done in Zaria, Nigeria documented a high sero-prevalence rate among pregnant women who drunk water from the well [25], this was not the case in the recent study done in Mexico [26] and in the present study. Contact with cat litter may pose another risk for *T. gondii* infection. However, in this study no significant association between *T. gondii* infection and a history of cat contact was found. The findings are consistent with studies done in Palestine [15], Turkey [21] and Nigeria [25]. Nevertheless, studies from Taiwan [27] and Ethiopia [6] showed a significant association between contact with cats and sero-prevalence of *T. gondii.* However, the risk of contracting *T. gondii* infection is not just the mere contact with cats but the way the cats’ litter is handled.

## Conclusions

Sero-prevalence of *T. gondii*-specific antibodies is high among pregnant women in Mwanza with a significant proportion of women at risk of contracting *T. gondii* infections. Advanced woman’s age, urban residence and being an employed or a business woman were the independent risk factors associated with the presence of *T. gondii* infections. Screening of *T. gondii* infections during antenatal care should be considered in Tanzania as the main strategy to prevent and minimize congenital toxoplasmosis.

## Competing interests

The authors declare that they have no competing interests.

## Authors’ contributions

BM, SEM, CM and UG participated in the design of the work. BM and AM participated in the collection of specimens and clinical data. UG analyzed the sample. BM, BRK, HDM, DM and SEM analyzed and interpreted the data. SEM and BRK wrote the first draft of the manuscript which was approved by all authors. All authors read and approved the final version of this manuscript.
